# Psilocybin improves novel object recognition in a rat model of Fragile X Syndrome through the modulation of the BDNF/TrkB signaling pathway

**DOI:** 10.1038/s41386-026-02361-x

**Published:** 2026-02-13

**Authors:** Fabrizio Ascone, Valeria Buzzelli, Francesca Mottarlini, Melania Di Trapano, Paolo Miglioranza, Alessandro Rava, Alessandro Feo, Flavio Spano, Marvin Hausman, Kiminobu Sugaya, Lucia Caffino, Fabio Fumagalli, Viviana Trezza

**Affiliations:** 1 Department of Science, Section of Biomedical Sciences and Technologies, University “Roma Tre”, Rome, Italy; 2 Department of Pharmacological and Biomolecular Sciences ‘Rodolfo Paoletti’, Università degli Studi di Milano, Milan, Italy; 3 Neuroendocrinology, Metabolism and Neuropharmacology Unit, IRCSS Fondazione Santa Lucia, Rome, Italy; 4 Nova Mentis Life Science Corp., Vancouver, BC Canada; 5 Burnett School of Biomedical Sciences, College of Medicine, University of Central Florida, Orlando, FL USA

**Keywords:** Autism spectrum disorders, Working memory

## Abstract

Fragile X Syndrome (FXS) is the most common inherited intellectual disability and a leading monogenic cause of autism spectrum disorder (ASD). As a synaptic disorder, FXS involves the loss of Fragile X messenger ribonucleoprotein 1 (FMRP), leading to abnormal dendrite development and immature dendritic spines. Serotonergic signaling, essential for neuronal development and circuit remodeling, has been implicated in ASD and related conditions, including FXS, raising the possibility that serotonergic modulation could ameliorate neurodevelopmental impairments. This study investigated the therapeutic potential of psilocybin, a serotonergic compound, in the validated *Fmr1-*^*Δ*^*exon 8* rat model of FXS. Psilocybin microdosing rescued deficits in NOR. Importantly, its benefits on recognition memory persisted despite pretreatment with the 5HT2AR antagonist, volinanserin, or the 5HT1AR antagonist, WAY-100635, indicating that classical serotonergic receptor activation is not required. In contrast, pretreatment with the TrkB receptor antagonist, ANA-12, abolished psilocybin’s effects, implicating BDNF/TrkB signaling as essential. At the molecular level, psilocybin normalized mature BDNF (mBDNF), increased TrkB, and restored downstream AKT signaling in the prefrontal cortex of *Fmr1-*^*Δ*^*exon 8* rats, pathways strongly linked to synaptic plasticity and cognitive function. These findings demonstrate that psilocybin rescues object recognition memory deficits in this rat model of FXS via BDNF/TrkB-AKT signaling rather than serotonergic receptor mechanisms. By dissociating therapeutic effects from hallucinogenic pathways, our results highlight psilocybin microdosing as a promising therapeutic strategy for neurodevelopmental disorders such as FXS and ASD.

## Introduction

Fragile X Syndrome (FXS) is the most common inherited form of intellectual disability and the leading monogenic cause of Autism Spectrum Disorder (ASD), affecting ~1 in 7000 males and 1 in 11,000 females [1]. It results from a CGG repeat expansion in the *FMR1* gene that silences expression of Fragile X Messenger Ribonucleoprotein 1 (FMRP) [2], a protein crucial for synaptic protein synthesis, mRNA transport, and activity-dependent synaptic plasticity. Its absence disrupts dendritic development and synaptic refinement, producing cognitive deficits, behavioral rigidity, and social dysfunction [2–4]. Nearly half of males and a significant portion of females with FXS meet criteria for ASD [5, 6].

Serotonergic dysregulation is a consistent feature of both ASD and FXS [7–9]. Serotonin regulates neurodevelopment and synaptic maturation, and aberrant serotonergic signaling has been consistently observed in individuals with ASD and FXS [8, 10]. Serotonergic drugs have been tested for their potential to improve social interaction and mood [11–14]. Psilocybin, a psychedelic tryptamine, is of particular interest: although its psychoactive effects are attributed to serotonin 2 A receptor (5HT2AR) agonism, its broader pharmacology suggests additional roles in neuroplasticity and cognition.

Psychedelics, including psilocybin, have recently been shown to promote neural plasticity by engaging brain-derived neurotrophic factor (BDNF) and its receptor TrkB [15, 16]. The BDNF/TrkB cascade is essential for synaptic development, learning, and memory [17, 18] and is disrupted in FXS and related neurodevelopmental disorders [19]. Given the functional interplay between serotonergic and neurotrophic systems, targeting BDNF/TrkB signaling in conjunction with serotonergic pathways may represent a new therapeutic strategy for FXS.

We previously reported that repeated low-dose psilocybin (“microdosing”) rescues object recognition deficits in the *Fmr1-*^*Δ*^*exon 8* rat [20], a validated model of FXS and ASD [21–23]. However, the mechanisms were unknown. While it is clear that psilocybin’s hallucinogenic effects depend on 5HT2AR activation [24, 25], it remains uncertain whether serotonergic receptors mediate its benefits on recognition memory. An alternative hypothesis is that psilocybin acts by restoring BDNF/TrkB-dependent plasticity.

In the present study, we investigated this hypothesis using pharmacological antagonism experiments and molecular analyses in *Fmr1-*^*Δ*^*exon 8* rats. We asked whether psilocybin’s benefits on short-term recognition memory require 5HT2A or 5HT1A receptor activation, and whether they depend on intact BDNF/TrkB signaling. Recognition memory was assessed with the novel object recognition (NOR) test, a widely used paradigm with prefrontal involvement. Molecular analyses examined Bdnf isoforms, mature BDNF (mBDNF), TrkB expression, and downstream AKT signaling in the prefrontal cortex (PFC).

By integrating behavioral and molecular approaches, we sought to determine whether psilocybin’s therapeutic effects in FXS derive from direct 5HT2A and 5HT1A receptor activation or involve neurotrophic mechanisms. Establishing this distinction has clinical importance: if efficacy can be achieved independently of serotonergic receptor activation, psilocybin or related compounds could provide benefits without hallucinogenic side effects, supporting microdosing as a safe and tolerable strategy for neurodevelopmental disorders.

## Materials and methods

### Experimental design and drugs

Wild-type (WT) and *Fmr1-*^*Δ*^*exon 8* rats were tested following the experimental design shown in Supplementary Fig. S1. Psilocybin was administered via oral gavage (0.1 mg/kg per os, p.o.) every other day for two weeks, from postnatal day (PND) 34 to PND 44. Animals were tested in the NOR test five days after the final administration to exclude any residual hallucinogenic effects and to capture enduring, post-acute outcomes [20]. Our dose regimen is consistent with a microdosing paradigm. Indeed, to provide quantitative support for our dosing choice, we applied standard FDA Km-based allometric scaling (rat Km = 6; human Km = 37) [26], yielding a human-equivalent dose (HED) of 0.016 mg/kg for our 0.1 mg/kg oral rat regimen — approximately 1.1 mg for a 70 kg adult — well within the empirically defined human microdose range of ~1–2.5 mg [27–29].

Experiment 1 investigated whether the recognition memory effects of psilocybin in *Fmr1-*^*Δ*^*exon 8* rats were dependent on 5HT2AR activation. The 5HT2AR antagonist volinanserin (M100907, Selleck Chemicals, USA) was dissolved in 5% Tween 80, 5% polyethylene glycol, and 90% saline, and administered intraperitoneally (i.p.) at the dose of 0.25 mg/kg 15 min before each psilocybin dose [30, 31]. This M100907 dose and pretreatment interval have been shown to suppress DOI- and LSD-induced head-twitch responses (HTRs) [31].

Experiment 2 examined the involvement of 5HT1AR in mediating the positive effects of psilocybin in the NOR test. The 5HT1AR antagonist WAY-100635 (MedChemExpress, USA) was dissolved in the same vehicle (VEH) and administered i.p. at the dose of 0.3 mg/kg 10 min prior to each psilocybin treatment [32].

Building on these results, Experiment 3 explored psilocybin-induced changes in the expression of 5HT1AR and 5HT2AR in the PFC by Western blot.

Experiments 4 and 5 tested the hypothesis that the beneficial effects of psilocybin in *Fmr1-*^*Δ*^*exon 8* rats might involve TrkB/BDNF signaling. The selective TrkB antagonist ANA-12 (Selleckchem, USA) was administered i.p. at the doses of 0.5 mg/kg (Experiment 4) and 1.0 mg/kg (Experiment 5), 3.5 h before each psilocybin administration. This pretreatment interval was selected based on ANA-12 pharmacokinetic profile, as the compound penetrates the brain within approximately 30 min but achieves maximal TrkB inhibition after 3–4 h, thereby ensuring effective receptor blockade during psilocybin exposure [33].

The timing of antagonist administration was further informed by pharmacokinetic data showing that oral psilocybin produces peak systemic psilocin levels and maximal HTRs within 15–30 min post-administration [34]. Thus, this schedule ensured overlap between the period of maximal TrkB inhibition and the peak of psilocybin’s central effects.

In Experiment 6, quantitative PCR (qPCR) was used to assess mRNA expression levels of total BDNF, as well as its *exons I*, *IV*, *VI*, and *long* isoforms, in PFC.

Experiment 7 further elucidated the molecular mechanisms underlying psilocybin’s effects by analyzing the expression of key neuroplasticity-related proteins (mBDNF, TrkB, pAKT_S473_, pAKT _S473_/AKT) via Western blot.

### Animals

Male WT (Charles River Laboratories, Italy) and *Fmr1-*^*Δ*^*exon 8* rats (Horizon Discovery, formerly SAGE Labs, USA) on a Sprague-Dawley background were housed in groups of three under controlled environmental conditions: temperature 20–21 °C, humidity 55–65%, 12:12 h light/dark cycle (lights on at 07:00 h). Behavioral experiments and key experimental procedures were conducted between 9:00 a.m. and 2:30 p.m. Sample sizes (*n*) are indicated in the figure legends and were based on previous studies and power analyses conducted using G*Power software. A single cohort of animals was used to perform biochemical analyses in Experiments 3, 6, and 7, to reduce variability and limit animal use. Conversely, five independent cohorts were employed for the behavioral assessments, with one cohort dedicated to each behavioral experiment to ensure proper counterbalancing and avoid potential confounding effects of repeated testing. Outliers were identified using the Grubbs’ test in GraphPad Prism 8 (GraphPad Software, USA). The behavioral experiments were scored in blind conditions using the Observer 3.0 software (Noldus Information Technology, The Netherlands).

Procedures were approved by the Italian Ministry of Health (authorization n. 988/2020-PR) and conducted in accordance with the ARRIVE guidelines, the Italian Legislative Decree No. 26/2014, and the European Community Directive 2010/63/EU.

### Novel object recognition (NOR) test

The test was conducted as previously described [35]. During the training phase, each rat was placed in an open field arena containing two identical objects (A1 and A2) and allowed to explore for 5 min. After a 30-min inter-trial interval, one familiar object (A3) and one novel object (B) were positioned in the exact location as during training. Each rat was then reintroduced to the arena for a 5-min test session.

Object exploration was scored when the animal was sniffing or touching the object with the nose and/or forepaws. The time spent exploring each object was recorded, and the discrimination index (DI) was calculated as the difference in time spent by each animal exploring the novel compared with the familiar object, divided by the total time spent exploring both objects in percentage.

To exclude potential confounding effects of locomotor or anxiety-like behavior, an independent cohort of *Fmr1-*^*Δ*^*exon 8* and WT rats was tested under identical housing, treatment, and experimental conditions in the open field and elevated plus-maze tasks (see Supplementary Information and Supplementary Fig. S2).

### Biochemical analysis

#### Brain samples collection

Rats were decapitated, and their brains were rapidly removed from the skull on a cold plate. The PFC was manually dissected from fresh brains immediately following decapitation as a tissue block comprising the prelimbic, infralimbic and anterior cingulate cortex [36, 37]. Dissections were performed under microscopic guidance within 2 min and stored at −80 °C until analysis. Western blot and real-time PCR experiments were performed using PFC samples collected at PND 50.

#### Western blot analysis

PFC tissues were homogenized in ice-cold buffer (0.32 M sucrose, 0.1 mM PMSF, 1 mM HEPES, 0.1 mM EGTA, pH 7.4) supplemented with protease (Roche-Merk Life Science, Italy) and phosphatase (Sigma-Aldrich-Merk Life Science, Italy) inhibitors. Homogenates were sonicated, and protein concentrations were determined using the Bradford Protein Assay (Bio-Rad, Italy), with bovine serum albumin as standard. Samples were stored at −20 °C until analysis.

Eight micrograms of proteins per sample were separated by SDS-PAGE (14% gels) and transferred to nitrocellulose membranes (Bio-Rad, Italy). Membranes were blocked (I-Block, Life Technologies, Italy) in TBS + 0.1% Tween-20 buffer, incubated with phospho-specific antibodies, then stripped and reprobed for corresponding total proteins. Conditions of the primary antibodies are reported in the Supplementary Materials. Detection was performed using Clarity Western ECL substrate (Bio-Rad, USA) and chemiluminescence captured with a ChemiDoc XRS+ system (Bio-Rad, USA). Band intensities were quantified using ImageLab software, normalized to β-actin (43 kDa), and expressed as fold change relative to WT controls. Representative immunoblots are presented in Figs. 2C and 5F; examples of uncropped blots are shown in Supplementary Fig. S3. Each sample was analyzed in duplicate or triplicate gels, and results were averaged using a correction factor to adjust for inter-gel variability, calculated as: correction factor gel B =average of (OD protein of interest/OD β-actin for each sample loaded in gel A)/(OD protein of interest/OD β-actin for the same sample loaded in gel B). Correction factor gel C = average of (OD protein of interest/OD β-actin for each sample loaded in gel A)/(OD protein of interest/OD β-actin for the same sample loaded in gel C) [38].

#### Real-time PCR

Total RNA was isolated using PureZol reagent (Bio-Rad, Italy), quantified by Nanodrop spectrophotometry, and stored at −20 °C [39].

RNA samples were treated with DNase I (Thermo Scientific, RNase-free) for 30 min at 37 °C to remove genomic DNA, followed by EDTA inactivation (10 min, 65 °C). Gene expression was assessed by one-step RT-qPCR (CFX384 Real-Time System, Bio-Rad) using the iScriptTM kit (Bio-Rad, Italy). Each sample was run in triplicate in a 384-well plate. Data were analyzed with the comparative threshold cycle (ΔΔCt) using 36B4 as an internal standard. RT-qPCR analyses were performed to evaluate the gene expression of *total Bdnf*, *Bdnf exon I*, *IV*, *VI, and Bdnf long*. Sequences of primers and probes are reported in the Supplementary Materials.

### Statistical analysis

Data are presented as mean ± standard error of the mean (SEM). Biochemical data were analyzed using two-way analysis of variance (ANOVA) to assess the effects of genotype and psilocybin. Behavioral data were analyzed using three-way ANOVA to evaluate the impact of genotype, psilocybin, and treatment with M100907, WAY-100635, or ANA-12. Tukey’s *post hoc* test was applied for multiple comparisons. Analyses were performed using GraphPad Prism version 8 (GraphPad Software, USA). Behavioral, biochemical (Western blot), and molecular (real-time PCR) data were analyzed by experimenters blinded to genotype and treatments.

## Results

### 5HT1AR and 5HT2AR do not mediate the effects of psilocybin on object recognition in *Fmr1-*^*Δ*^*exon* 8 rats

**Fig. 1:**
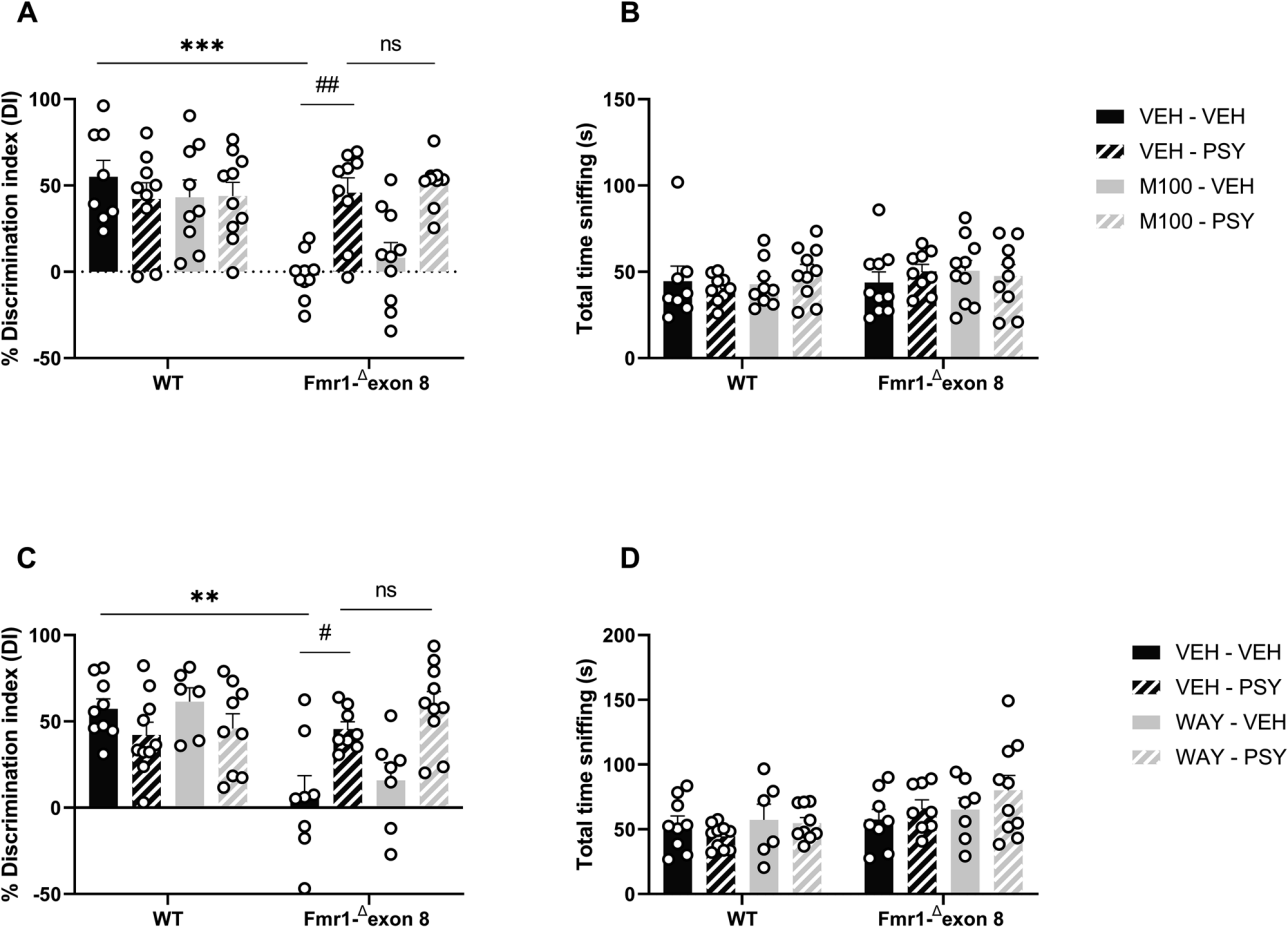
5HT2AR and 5HT1AR antagonism does not block the beneficial effects of psilocybin on novel object discrimination in *Fmr1-*^*Δ*^*exon 8* rats. Systemic administration of the 5HT2AR selective antagonist M100 (0.25 mg/kg, i.p.) did not counteract the positive effects of psilocybin (PSY) on object recognition in *Fmr1-*^*Δ*^*exon 8* rats (**A**). Total object exploration time during testing was unaffected by treatment across genotypes (**B**) (WT-VEH/VEH = 8, WT-VEH/PSY = 9, WT-M100/VEH = 9, WT-M100/PSY = 10, *Fmr1-*^*Δ*^*exon 8-*VEH/VEH = 10, *Fmr1-*^*Δ*^*exon 8-* VEH/PSY = 9, *Fmr1-*^*Δ*^*exon 8-* M100/VEH = 10*, Fmr1-*^*Δ*^*exon 8-*M100/PSY = 9). Similarly, administration of the 5HT1AR selective antagonist WAY (0.3 mg/kg, i.p.) failed to prevent the recognition memory-enhancing effects of psilocybin in *Fmr1-* ^*Δ*^*exon 8* rats (**C**), and had no impact on total object exploration time (**D**) (WT-VEH/VEH = 9, WT-VEH/PSY = 10, WT-WAY/VEH = 6, WT-WAY/PSY = 9, *Fmr1-*^*Δ*^*exon 8-*VEH/VEH = 8, *Fmr1-*^*Δ*^*exon 8-* VEH/PSY = 8, *Fmr1-*^*Δ*^*exon 8-* WAY/VEH = 7*, Fmr1-*^*Δ*^*exon 8-*WAY/PSY = 10). Data represent mean ± SEM, ^**^p < 0.01, ^***^p < 0.001 vs WT-VEH/VEH group, ^#^*p* < 0.05, ^##^*p* < 0.01, vs *Fmr1-*^*Δ*^*exon 8*-VEH/VEH group; (three-way ANOVA followed by Tukey’s *post hoc* test).

As expected [20], psilocybin improved performance in the NOR task, and this effect was not secondary to changes in locomotor activity or emotionality (see the results of the open field and elevated plus-maze tests in Supplementary information). Pretreatment with the 5HT2AR antagonist M100907 (M100, 0.25 mg/kg) failed to block psilocybin’s rescue of object recognition in *Fmr1-*^*Δ*^*exon 8* rats (main effects of genotype: *F*_1,66_ = 12.57, *p* < 0.001; psilocybin: *F*_1,66_ = 11.95, *p* < 0.001; genotype × psilocybin: *F*_1,66_ = 20.40, *p* < 0.001; all other effects n.s.; Fig. 1A). Similarly, the 5HT1AR antagonist WAY-100635 (WAY, 0.3 mg/kg) failed to alter psilocybin’s effect (genotype: *F*_1,59_ = 11.43, *p* < 0.01; psilocybin: *F*_1,59_ = 4.95, *p* < 0.05; genotype × psilocybin: *F*_1,59_ = 23.31, *p* < 0.001; all other effects n.s.; Fig. 1C). Total objects exploration time was unchanged in both conditions (Figs. 1B, D and Supplementary Table 1), indicating that psilocybin’s enhancement of object recognition was not due to altered exploratory behavior. These results show that psilocybin’s pro-cognitive effects in the NOR task are independent of direct 5HT2AR and 5HT1AR activation.

For all statistical details, see Supplementary Table 1.

### Psilocybin selectively modulates 5HT1AR but not 5HT2AR protein levels in the PFC

**Fig. 2:**
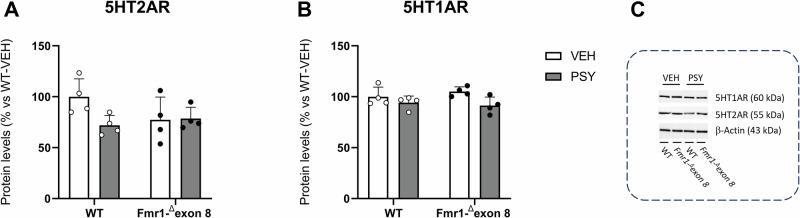
Western blot analysis of 5HT2AR and 5HT1AR protein levels in the PFC of WT and *Fmr1-*^*Δ*^*exon 8* rats following psilocybin treatment. Protein levels of 5HT2AR (**A**) and 5HT1AR (**B**). **C** shows representative immunoblots related to the expression levels of 5HT2AR (55 kDa) and 5HT1AR (60 kDa) in the PFC lysates. Each sample was analyzed in triplicate gels, and results were averaged after correction for inter-gel variability. Densitometric values were normalized to β-actin and expressed as fold change relative to WT vehicle-treated controls (WT-VEH). (WT-VEH = 4, *Fmr1-*^*Δ*^*exon 8*-VEH = 4, WT-PSY = 4, *Fmr1-*^*Δ*^*exon 8*-PSY = 4). Data represent means ± SEM (two-way ANOVA followed by Tukey’s *post hoc* test).

No significant main effects of genotype, treatment, or genotype × treatment interaction were found on 5HT2AR expression (Fig. 2A). In contrast, 5HT1AR levels showed a significant main effect of treatment (psilocybin: *F*_1,12_ = 6.819, *p* < 0.05; all other effects n.s.; Fig. 2B), although *post hoc* comparisons did not reveal significant differences between individual groups.

These results show that repeated psilocybin administration produces a modest treatment effect on 5HT1AR expression independent of genotype, while 5HT2AR levels remain unchanged in the PFC.

For all statistical details, see Supplementary Table 2.

### TrkB receptor mediates the effects of psilocybin on object recognition in *Fmr1-*^*Δ*^*exon 8* rats

**Fig. 3:**
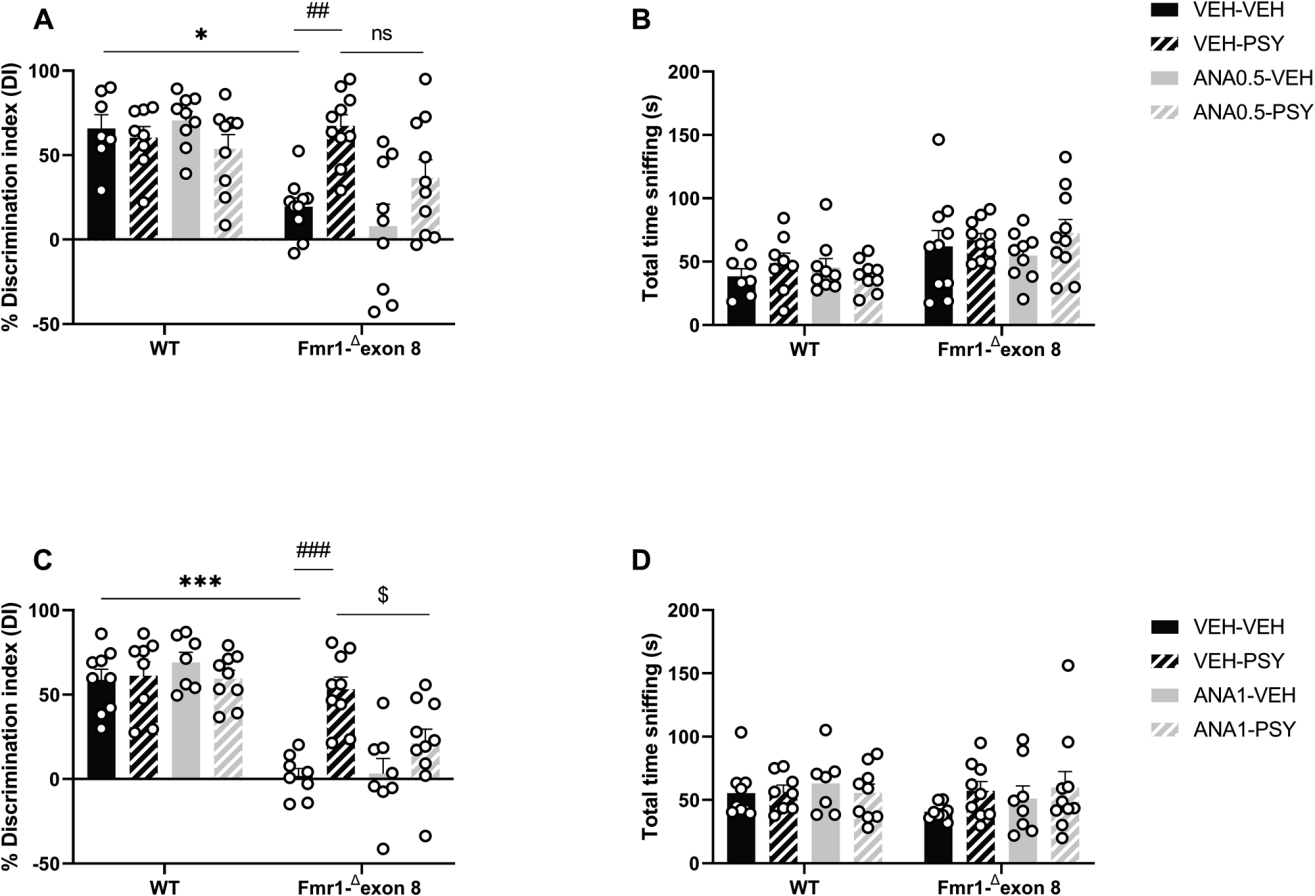
TrkB antagonism prevents the beneficial effects of psilocybin on novel object discrimination in *Fmr1-*^*Δ*^*exon 8* rats. Systemic administration of the selective TrkB antagonist ANA-12 at the dose of 0.5 mg/kg (i.p., ANA 0.5) did not prevent the recognition memory-enhancing effects of psilocybin (0.1 mg/kg, p.o.) in *Fmr1-*^*Δ*^*exon 8* rats, as measured by the novel object recognition (NOR) test (**A**). Total object exploration time during testing was unaffected across all groups (**B**). (WT-VEH/VEH = 7, WT-VEH/PSY = 8, WT-ANA0.5/VEH = 9, WT-ANA0.5/PSY = 9, *Fmr1-*^*Δ*^*exon 8-*VEH/VEH = 10, *Fmr1-*^*Δ*^*exon 8-* VEH/PSY = 10, *Fmr1-*^*Δ*^*exon 8-* ANA0.5/VEH = 9*, Fmr1-*^*Δ*^*exon 8-* ANA0.5/PSY = 10). In contrast, a higher dose of ANA-12 (1.0 mg/kg, i.p., ANA 1) effectively blocked psilocybin’s pro-cognitive effects in *Fmr1-*^*Δ*^*exon 8* rats (**C**), while still not altering total object exploration time (**D**). (WT-VEH/VEH = 9, WT-VEH/PSY = 8, WT-ANA1/VEH = 7, WT-ANA1/PSY = 9, *Fmr1-*^*Δ*^*exon 8-*VEH/VEH = 8, *Fmr1-*^*Δ*^*exon 8-* VEH/PSY = 9, *Fmr1-*^*Δ*^*exon 8-* ANA1/VEH = 8*, Fmr1-*^*Δ*^*exon 8-* ANA1/PSY = 10). Data represent mean ± SEM, ^***^p < 0.001 vs WT-VEH/VEH group, ^###^*p* < 0.001 vs *Fmr1-*^*Δ*^*exon 8*-VEH/VEH group; ^$^*p* < 0.05, vs *Fmr1-* ^*Δ*^*exon 8*-VEH/PSY (three-way ANOVA followed by Tukey’s *post hoc* test).

Given prior evidence that psilocybin also acts via TrkB receptor activation [16], we investigated whether TrkB signaling contributes to the recognition memory-enhancing effects of psilocybin in *Fmr1-*^*Δ*^*exon 8* rats. At the lower dose (0.5 mg/kg), the TrkB antagonist ANA-12 did not modify psilocybin’s positive effect on object recognition (genotype: *F*_1,64_ = 24.23, *p* < 0.001; psilocybin: *F*_1,64_ = 5.004, *p* < 0.05; genotype × psilocybin: *F*_1,64_ = 16.77, *p* < 0.001; all other effects n.s.; Fig. 3A). Total object exploration time during test remained unaffected (Fig. 3B). Conversely, the higher dose of ANA-12 (1.0 mg/kg) abolished psilocybin’s beneficial effect on object recognition (genotype: *F*_1,60_ = 71.62, *p* < 0.001; psilocybin: *F*_1,60_ = 9.696, *p* < 0.01; genotype × psilocybin: *F*_1,60_ = 14.79, *p* < 0.001; ANA-12 × psilocybin: *F*_1,60_ = 5.156, *p* < 0.05; all other effects n.s.; Fig. 3C), leaving total exploration time unchanged (Fig. 3D). These results support a mechanistic link between psilocybin’s pro-cognitive actions in the NOR task and BDNF/TrkB pathway engagement.

For all statistical details, see Supplementary Table 3.

### Psilocybin enhances cortical *Bdnf* transcription in WT, but not in *Fmr1-*^*Δ*^*exon 8* rats

**Fig. 4:**
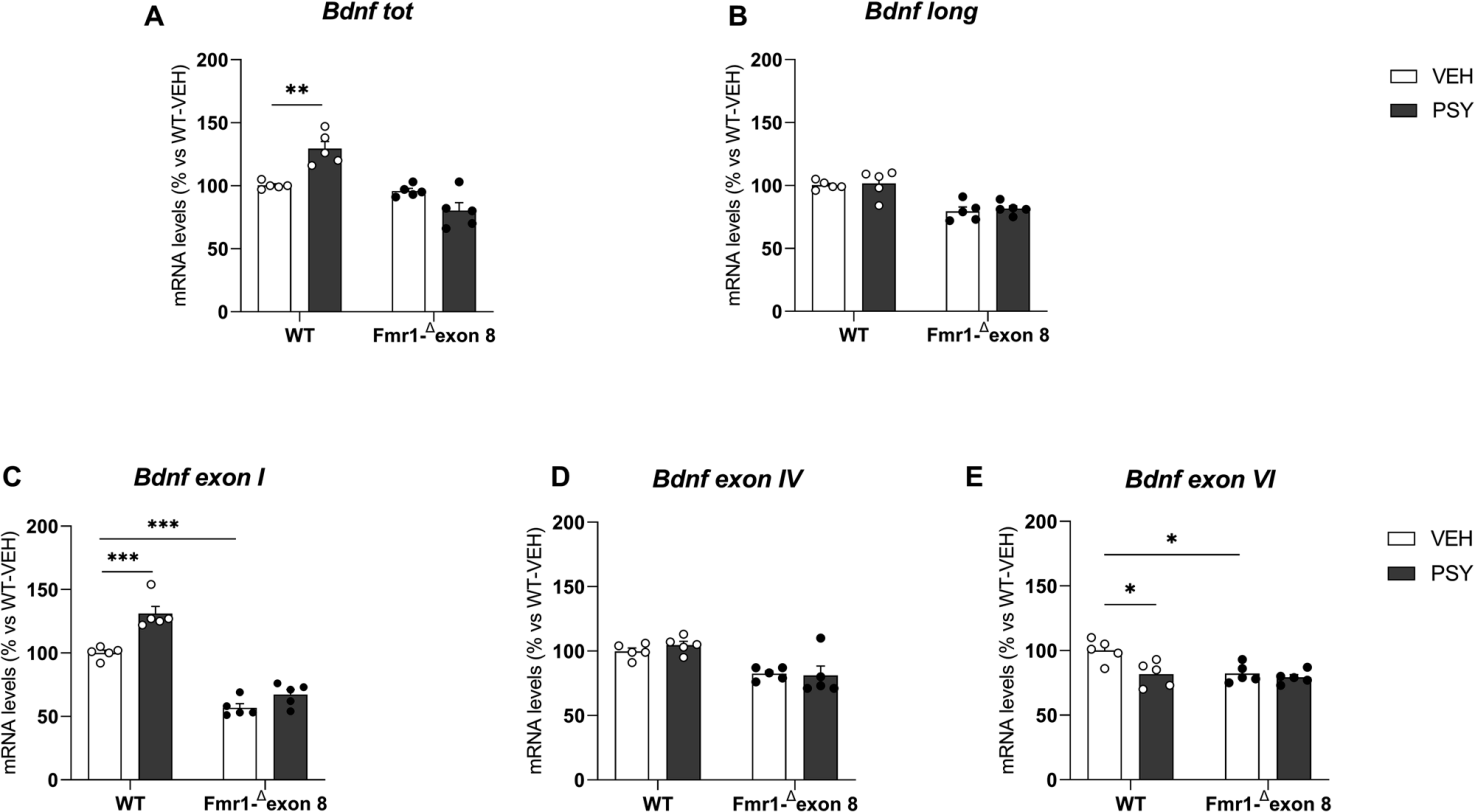
Effect of repeated administration of psilocybin on *Bdnf* mRNA levels in the PFC. Data represent the mRNA levels for total *Bdnf* (**A**), *Bdnf long* (**B**), *Bdnf exon I* (**C**), *Bdnf exon IV* (**D**) and *Bdnf exon VI* (**E**) in the PFC expressed as a percentage of VEH-treated rats. (WT-VEH = 5, *Fmr1-*^*Δ*^*exon 8*-VEH = 5, WT-PSY = 5, *Fmr1-*^*Δ*^*exon 8*-PSY = 5). Data represent means ± SEM, *p < 0.05, **p < 0.01 and ***p < 0.001 vs WT-VEH group (two-way ANOVA followed by Tukey’s *post hoc* test).

Psilocybin significantly increased *total Bdnf* mRNA levels in WT rats, whereas no effect was observed in *Fmr1-*^*Δ*^*exon 8* animals, indicating a genotype-dependent impairment in transcriptional responsiveness (genotype: *F*_1,16_ = 35.65, *p* < 0.0001; genotype × psilocybin: *F*_1,16_ = 24.91, *p* = 0.0001; all other effects n.s.; Fig. 4A). In contrast, gene expression of *Bdnf long*, the pool of transcript with the long polyadenylation site at 3’UTR [40], was significantly affected by genotype only (genotype: *F*_1,16_ = 38.69; *p* < 0.0001; all other effects n.s.; Fig. 4B), suggesting that this transcript variant is not responsive to psilocybin. Conversely, a robust treatment effect was observed for *Bdnf exon I* mRNA, an activity-dependent transcript located in the soma [41]. Indeed, psilocybin markedly upregulated *exon I* expression in WT rats, while *Fmr1-*^*Δ*^*exon 8* rats exhibited significantly reduced baseline levels and failed to respond to treatment (genotype: *F*_1,16_ = 174.6, *p* < 0.0001; psilocybin: *F*_1,16_ = 26.14, *p* = 0.0001; genotype × psilocybin: *F*_1,16_ = 6.471, *p* < 0.05; Fig. 4C), reinforcing the notion of disrupted activity-dependent transcriptional regulation in the mutant genotype. *Bdnf exon IV* mRNA levels, an activity-dependent transcript targeted in the proximal dendrites [42], were altered only depending on the genotype (genotype: *F*_1,16_ = 22.31, *p* < 0.001; all other effects n.s.; Fig. 4D). Interestingly, *Bdnf exon VI* expression, an activity-dependent transcript targeted to the distal dendrites [43], was significantly reduced by psilocybin in WT rats compared to VEH controls, with *Fmr1-*^*Δ*^*exon 8* rats exhibiting overall lower *Bdnf exon VI* expression levels compared to WT-VEH (genotype: *F*_1,16_ = 8.062, *p* < 0.05; psilocybin: *F*_1,16_ = 8.707, *p* = 0.01; all other effects n.s.; Fig. 4E).

Collectively, these findings demonstrate that psilocybin elicits a selective upregulation of total *Bdnf* and *exon I* transcripts in the PFC of WT rats, an effect that is absent in *Fmr1-*^*Δ*^*exon 8* animals. This suggests that FMRP is required for psilocybin-induced activation of specific *Bdnf* isoforms, pointing to impaired neuroplasticity-related transcriptional mechanisms in this rat model of FXS.

For all statistical details, see Supplementary Table 4.

### Psilocybin enhances mBDNF-TrkB and AKT signaling in the PFC of *Fmr1-*^*Δ*^*exon 8* rats

To investigate whether psilocybin modulates the BDNF/TrkB intracellular pathway, we assessed the expression of mBDNF, its high-affinity receptor TrkB, and one of its downstream signaling effector AKT by assessing both its levels of phosphorylation at Ser473 (pAKT_S473_) and expression in the PFC of WT and *Fmr1-*^*Δ*^*exon 8* rats.

**Fig. 5:**
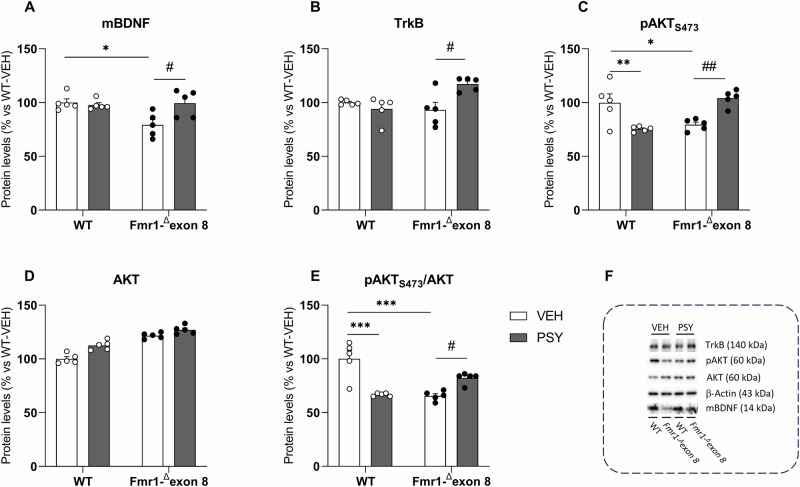
Western blot analysis of mBDNF, TrkB and AKT in the PFC of WT and *Fmr1-*^*Δ*^*exon 8* rats following repeated oral psilocybin treatment. **A** mBDNF; **B** TrkB; **C** phosphorylated AKT (pAKT_S473_); **D** total AKT; **E** pAKT _S473_/AKT ratio measured in the whole homogenate of the PFC. Each sample was analyzed in duplicate gels, and results were averaged after correction for inter-gel variability. Densitometric values were normalized to β-actin and expressed as a percentage relative to vehicle-treated controls (WT-VEH). **F** shows representative immunoblots for TrkB (140 kDa), pAkt (ser473) and Akt (60 kDa), β-actin (43 kDa) and mBDNF (14 kDa). (WT-VEH = 5, *Fmr1-*^*Δ*^*exon 8*-VEH = 5, WT-PSY = 5, *Fmr1-*^*Δ*^*exon 8*-PSY = 5). Data represent means ± SEM, *p < 0.05, **p < 0.01 and ***p < 0.001 vs WT-VEH group; ^#^*p* < 0.05, ^##^*p* < 0.01 vs *Fmr1-*^*Δ*^*exon 8*-VEH group (two-way ANOVA followed by Tukey’s *post hoc* test).

Psilocybin did not alter mBDNF levels in WT rats but normalized its levels in *Fmr1-*^*Δ*^*exon 8* rats, which were significantly lower than those observed in WT controls (genotype: *F*_1,16_ = 5.103, *p* < 0.05; genotype × psilocybin: *F*_1,16_ = 6.946, *p* < 0.05; all other effects n.s.; Fig. 5A).

In line with mBDNF levels, psilocybin did not change TrkB expression in WT animals while it increased TrkB protein levels in *Fmr1-*^*Δ*^*exon 8* rats (interaction genotype × psilocybin: *F*_1,16_ = 10.36, *p* < 0.01; all other effects n.s.; Fig. 5B).

Psilocybin reduced pAKT_S473_ levels in WT animals while it normalized the reduced phosphorylation of AKT observed in VEH-treated *Fmr1-*^*Δ*^*exon 8* rats (interaction genotype × psilocybin: *F*_1,16_ = 27.73, *p* < 0.001; all other effects n.s.; Fig. 5C).

Although AKT expression was altered by the genotype and treatment, no significant changes in the interaction between the two variables were observed (main effects of genotype: *F*_1,16_ = 103.5, *p* < 0.001; psilocybin: *F*_1,16_ = 25.88, *p* < 0.001; all other effects n.s.; Fig. 5D). As an indirect index of activation of TrkB downstream signaling, we measured the pAKT/AKT ratio. Psilocybin significantly reduced this ratio in WT animals and reversed the reduction observed in VEH-treated *Fmr1-*^*Δ*^*exon 8* rats, restoring levels comparable to WT controls (genotype: *F*_1,16_ = 5.286, *p* < 0.05; genotype × psilocybin: *F*_1,16_ = 37.39, *p* < 0.001; all other effects n.s.; Fig. 5E).

Collectively, these findings indicate that psilocybin selectively enhances mBDNF-mediated signaling and its downstream intracellular pathways in *Fmr1-*^*Δ*^*exon 8* rats.

For all statistical details, see Supplementary Table 5.

## Discussion

Psychedelics, particularly psilocybin, are gaining increasing attention as treatments for psychiatric and neurodevelopmental disorders [20, 44–49]. Beyond their efficacy in anxiety and depression [50–52], recent work suggests that psychedelics may also enhance social behavior and cognitive performance, domains disrupted in ASD and FXS [14, 53]. Anecdotal reports further indicate that ASD individuals may experience lasting benefits, such as improved self-understanding and management of autistic traits [54]. Together, these findings support systematic investigation of psilocybin’s mechanisms in preclinical models of neurodevelopmental disorders.

Although psilocybin exerts its primary effects via activation of serotonergic 5HT2AR, the underlying mechanisms by which it may benefit ASD-related conditions remain poorly understood. Here, we confirmed that psilocybin rescues object recognition deficits in *Fmr1-*^*Δ*^*exon 8* rats, consistent with our prior findings [20]. Importantly, this benefit persisted despite pretreatment with selective antagonists of 5HT2AR or 5HT1AR, indicating that direct activation of these receptors is not required for psilocybin’s effects on recognition memory. Conversely, blockade of TrkB signaling abolished the effect, underscoring the necessity of BDNF-TrkB signaling in mediating psilocybin’s impact on object recognition.

This dissociation adds to a growing body of work showing that the therapeutic and hallucinogenic effects of psychedelics are not inseparable. While antagonism of 5HT2AR reliably prevents acute psychotomimetic responses, such as head-twitch behavior in rodents, it does not consistently block psilocybin-induced plasticity or sustained behavioral benefits [16, 25, 55]. For instance, Shao et al. [50] recently demonstrated that 5HT2ARs in pyramidal tract neurons are required for psilocybin’s anxiolytic and stress-alleviating effects in mice, underscoring a cell–type–specific contribution of cortical 5HT2AR signaling to its long-term impact on emotional reactivity. Conversely, Moliner et al. [16] reported that the effects of psychedelics on neurotrophic signaling, structural plasticity, and antidepressant-like behaviors in mice depend on TrkB binding and the enhancement of endogenous BDNF signaling, but are independent of 5HT2AR activation. Although our data suggest that classical membrane-bound 5HT2AR activation is unnecessary to mediate psilocybin’s benefits on object recognition, they do not exclude a contribution from intracellular 5HT2AR signaling, as recently suggested [56]. Indeed, it has been proposed that intracellular 5HT2AR activation contributes to neuroplastic effects of psychedelics [56], although the intracellular 5HT2AR hypothesis remains an evolving area of research. Future studies are therefore needed to disentangle the respective contributions of intracellular versus membrane-associated 5HT2AR receptor pools to psilocybin’s long-term effects.

Finally, although some authors argue that subjective psychedelic experiences may be necessary for therapeutic outcomes [57], growing evidence of 5HT2AR-independent antidepressant responses in the absence of hallucinogenic effects, together with our findings, support the possibility that psilocybin can engage distinct mechanisms to enhance cognition. This distinction is clinically important, as it opens the door to strategies that capture the therapeutic benefits of psychedelics while avoiding hallucinogenic liabilities.

The safety profile of the dosing regimen used in this study further supports this potential. Repeated administration of low-dose psilocybin was well tolerated in *Fmr1-*^*Δ*^*exon 8* rats, with no overt signs of adverse effects, including the absence of the “*wet dog shake*” response, a commonly used rodent behavioural correlate of hallucinogenic activity. Nonetheless, the lack of this behavioural marker does not exclude the possibility of more subtle central effects that may not be detectable using this measure alone. These findings align with reports that sub-perceptual doses in rodents produce beneficial effects without overt behavioral toxicity, supporting the feasibility of microdosing strategies for sensitive populations such as children and adolescents with neurodevelopmental disorders [58].

Within this context, elucidating the molecular mechanisms involved in psilocybin’s beneficial effects is crucial. Emerging evidence suggests that the therapeutic effects of psilocybin may depend, at least partially, on the modulation of the BDNF/TrkB pathway. Although psilocybin is not a direct TrkB agonist, it may act allosterically, enhancing the effects of BDNF released at active synapses [16]. Consistent with this model, previous work demonstrated that TrkB inhibition with ANA-12 abolishes psychedelic-induced dendritic growth [15]. Our findings align with an involvement of BDNF in psilocybin action, since the positive effects of psilocybin on object recognition in *Fmr1-*^*Δ*^*exon 8* rats were blocked by ANA-12, particularly at the higher dose (1.0 mg/kg). However, the upstream mechanisms linking psilocybin exposure to TrkB engagement were not directly addressed in the present study.

Importantly, psilocybin’s mechanism is unlikely to operate through an “either/or” process. Rather, it likely engages a cascade of interconnected signaling events. Activation of 5HT2AR may initiate upstream processes, including cortical burst firing, glutamate release, and immediate early gene induction, that converge on neurotrophin release and TrkB activation [15, 58, 59]. Recent evidence further demonstrates that serotonergic signaling via 5HT2AR can directly modulate TrkB receptor function through heteroreceptor complex formation [60], providing a mechanistic link between serotonergic and neurotrophic signaling pathways.

Taken together, our findings suggest that by the time psilocybin’s behavioral benefits emerge in this FXS model, engagement of the BDNF-TrkB pathway becomes indispensable, whereas isolated activation of 5HT2A/1A receptors is not sufficient to sustain the observed beneficial effects in the absence of TrkB signaling.

At the molecular level, our results reveal that psilocybin restores impaired neurotrophic signaling in *Fmr1-*^*Δ*^*exon 8* rats. Indeed, these animals exhibit reduced transcription of two of the major *Bdnf exons*, *exon I* and *exon VI*. The various isoforms of the complex *Bdnf* gene are under the control of distinct promoters and serve specialized functions in regulating BDNF expression and subcellular localization, thereby influencing whether BDNF primarily acts in the soma or dendrites [61]. E*xon I* is highly regulated by neuronal activity and calcium signaling within the soma [62], whereas *exon VI*, which is also activity-dependent, regulates structural and functional plasticity at distal dendrites [61]. Since *exon I*–containing mRNAs are important in activity-induced transcription in response to synaptic inputs, and can contribute to synaptic strengthening [41], their reduced transcription in *Fmr1-*^*Δ*^*exon 8* rats might contribute, at least in part, to dysfunction in the cortical-dependent behaviors, such as executive functions and object recognition [63–67]. Similarly, reduced *exon VI* transcription in these animals suggests a broader downregulation of PFC neuronal activation. The reduced expression of both transcripts, segregated in specific cellular compartments [61], may reflect a generalized impairment of cortical plasticity in *Fmr1-*^*Δ*^*exon 8* rats. Interestingly, in WT animals, psilocybin differently modulated the expression of the different exons, suggesting specific sub-mechanisms triggering BDNF response. Specifically, psilocybin enhanced *total Bdnf* gene expression, likely through activity-dependent upregulation of *exon I*. Since increased *exon I* expression is associated with adaptive coping responses to stress [68] as well as with enhanced mushroom spine density and size [62], these findings might suggest that psilocybin facilitates functional and structural plasticity under WT conditions. Conversely, the reduced expression of *exon VI* in WT rats may indicate reduced transcription of the specific neurotrophin pool at distal dendrites [43]. This reduction of *Bdnf exon VI* transcription in the PFC following psilocybin treatment in WT animals may represent a transient suppression of dendritically localized, activity-dependent BDNF signaling, facilitating a temporary weakening or pruning of existing synaptic connections that enables large-scale remodeling and reorganization of cortical microcircuits, an adaptive “reset” phase preceding the subsequent stabilization of newly configured synapses and network states [69]. More importantly, psilocybin rescued downstream deficits in *Fmr1-*^*Δ*^*exon 8* rats by normalizing mBDNF protein levels, increasing TrkB receptor expression, and restoring phosphorylation of AKT. This pattern suggests that psilocybin enhances the effectiveness of existing BDNF-TrkB signaling rather than driving new transcription, reactivating neuroplastic pathways that are otherwise blunted in FXS.

AKT signaling is a critical downstream effector of BDNF-TrkB and has been consistently implicated in the pathophysiology of FXS. Reduced activation of AKT has been documented in FXS patients, but not in patients with intellectual disability associated with obstetric complications, suggesting a role for this signaling pathway in the pathophysiology of FXS [70]. In the *Fmr1*-KO mouse model, both hyperactivation [71, 72] and hypoactivation [73] of the PI3K/AKT/mTOR pathway in the hippocampus and cortex have been reported. These seemingly divergent findings highlight the need for further investigation but consistently support the hypothesis that dysregulation of this pathway plays a critical role in FXS.

By reinstating AKT activation in *Fmr1-*^*Δ*^*exon 8* rats, psilocybin corrected a key molecular deficit linked to impaired synaptic plasticity and cognition. Interestingly, psilocybin modestly decreased the pAKT/AKT ratio in WT animals, perhaps reflecting a homeostatic mechanism to prevent excessive activation. Together, these data suggest that psilocybin selectively restores signaling imbalances in the FXS brain while maintaining stability in normal circuits.

Our results also align with earlier findings demonstrating altered BDNF and TrkB expression in Fmr1-KO mice and in neuronal cultures derived from these models [74, 75]. Moreover, exogenous enhancement of BDNF levels improved cognitive performance in Fmr1-KO mice, even in the absence of detectable baseline deficits in BDNF expression [76], underscoring the central role of this neurotrophin in supporting synaptic plasticity. The convergence of psilocybin-induced increases in BDNF and normalization of AKT activity provides a mechanistic explanation for its behavioral effects. Given the complex regulation of *Bdnf* isoforms [77], the selective changes we observed in exon-specific transcripts further suggest that psilocybin may fine-tune BDNF signaling at distinct subcellular compartments. However, since we have not provided a causal demonstration of their localization and direct function, this requires further study.

The parallels with ketamine provide additional insight. Despite acting on different primary targets, both psilocybin and ketamine rapidly engage BDNF-TrkB signaling [78] and produce long-lasting behavioral improvements at low, sub-perceptual doses. Prior work from our team has shown that ketamine’s antidepressant effects are separable from its reinforcing properties and are tightly linked to dynamic modulation of BDNF signaling [39, 79]. Our current findings suggest that psilocybin shares this capacity, pointing to BDNF-TrkB signaling as a convergent pathway across distinct classes of rapid-acting neuroplasticity-promoting agents. This convergence highlights the potential of targeting downstream neurotrophic signaling rather than receptor-specific pathways for therapeutic innovation.

Some limitations warrant consideration. First, our experiments were performed exclusively in male rats. Given the sex-specific differences documented in preclinical models of ASD [80] and the fact that FMRP loss can produce divergent effects in males and females [81], this represents an important limitation. However, a parallel cohort of female *Fmr1-*^*Δ*^*exon 8* rats is currently being tested to directly assess potential sex-dependent responses to psilocybin, ensuring that future work addresses this translationally relevant dimension.

Second, we focused on recognition memory using the NOR task, which involves prefrontal function, but it will be important to assess whether psilocybin also benefits other ASD-relevant domains such as social interaction, communication, and repetitive behaviors. Finally, while our data clearly implicate BDNF-TrkB-AKT signaling in mediating psilocybin’s effects, the specific cellular populations and circuit-level mechanisms remain to be elucidated. In particular, our molecular analyses were performed on PFC tissue, precluding cell-type–specific or circuit-resolved conclusions and not addressing potential contributions from other brain regions. Future studies employing regionally expanded, cell-type–specific, and circuit-level approaches will therefore be required to further refine the mechanistic interpretation of our findings.

In conclusion, we demonstrate that psilocybin rescues the object recognition deficits in *Fmr1-*^*Δ*^*exon 8* rats through reactivation of BDNF-TrkB-AKT signaling, independently of direct activation of 5HT2A and 5HT1A receptors (see Supplementary Fig. S4). By separating therapeutic from hallucinogenic effects, these findings support the hypothesis that low-dose psilocybin engages BDNF-TrkB-AKT signaling pathways implicated in neurodevelopmental disorders. At the same time, they underscore the need for careful preclinical and clinical evaluation before any conclusions regarding therapeutic applicability or safety can be drawn. More broadly, our results highlight BDNF-TrkB signaling as a shared pathway for therapeutic plasticity across diverse compounds, strengthening the rationale for interventions that modulate neurotrophic signaling to restore cognition in conditions characterized by impaired synaptic function.

## Supplementary information

Supplementary materials

## Data Availability

The data that support the findings of this study are available from the corresponding author upon reasonable request.
