# Supplementary material for: Psilocybin improves novel object recognition in a rat model of Fragile X Syndrome through the modulation of the BDNF/TrkB signaling pathway
Source: Neuropsychopharmacology. 2026 Feb 13;51(8):1454–63. doi: 10.1038/s41386-026-02361-x (PMC13291337; doi:10.1038/s41386-026-02361-x)
Supplement: Supplementary file 1 — Supplementary materials [file 41386_2026_2361_MOESM1_ESM.pdf]

Open field test

The open field apparatus consisted of a square arena (40 × 40 × 60 cm; length × width × height). Each animal was individually placed in the central zone of the apparatus and allowed to explore for 5 minutes. This assessment was performed during the habituation phase of the novel object recognition (NOR) task in a separate cohort of animals tested under identical housing, psilocybin treatment, and experimental conditions as those described for the main NOR experiment. After each session, the apparatus was cleaned with 70% ethanol. Each session was recorded with a camera positioned above the apparatus for subsequent behavioral analysis using The Observer 3.0 software (Noldus Information Technology, The Netherlands). The following parameters were analyzed: the number of crossings (a grid dividing the arena into equally sized squares, 8 × 8 cm, was projected onto the recordings, and the number of line crossings made by the animal was manually scored), and the time spent in the central (15 × 15 cm) and peripheral (within 5 cm from the walls) zones of the arena.

Elevated Plus Maze Test

The elevated plus maze apparatus comprised two open (50 × 10 × 40 cm; length × width × height) and two closed arms (50 × 10 × 40 cm; length × width × height) that extended from a common central platform (10 × 10 cm). Rats were individually placed on the central platform of the maze for 5 min. Each 5-min session was recorded with a camera positioned above the apparatus for subsequent behavioral analysis carried out by an observer, unaware of animal treatment and genotype, using the Observer 3.0 software (Noldus Information Technology, NL). The following parameters were analyzed:

- % Time spent in the open arms (% TO): (seconds spent on the open arms of the maze/ seconds spent on the open arms of the maze + seconds spent on the closed arms of the maze) × 100;
- number of total entries (frequency of entries into closed and open arms and the center of the maze).

Western blot analysis

Primary antibodies included: anti-5HT2A (1:3000, Sigma-Aldrich), anti-5HT1A (1:3000, GeneTex), anti-mBDNF (1:500, Icosagen), anti-TrkB (1:1000, Cell Signaling Technology), anti-Akt (1:1000, Cell Signaling Technology), anti phospho-Akt Ser473 (1:1000, Cell Signaling Technology), and anti-β-actin (1:5000, Sigma-Aldrich). After washing, membranes were incubated with the horseradish peroxidase-conjugated secondary antibody (Anti-Mouse, Cell Signaling Technology, 1:2000; Anti-Rabbit, Cell Signaling Technology, 1:2000) in 2 % milk in TBS-T for 1 h.

Real-time PCR

Primers and probe for *Bdnf exons IV* and *VI* were purchased from Applied Biosystem (*Bdnf exon IV*: ID Rn01484927_m1 and *Bdnf exon VI*: ID Rn01484928_m1; *Bdnf long*: ID Rn02531967_s1). Primers for *total Bdnf*, *Bdnf exon I*, and *36B4* were purchased from Eurofins MWG-Operon and the

primer efficiency was experimentally set up to identify the optimal concentration. Their sequences are shown below.

- *Total Bdnf*: forward primer 5′-AAGTCTGCATTACATTCCTCGA-3′, reverse primer GTTTTCTGAAAGAGGGACAGTTTAT-3′, probe 5′-TGTGGTTTGTTGCCGTTGCCAAG-3′;
- *Bdnf exon I*: forward primer 5′-GGGAGACGAGATTTTAAGACACTG-3′, reverse primer GTCATCACTCTTCTCACCTGG-3′, probe 5′-TTGTGGCTTTGCTGTCCTGGAGA-3′;
- *36B4*: forward primer 5′-TTCCCACTGGCTGAAAAGGT-3′, reverse primer 5′-CGCAGCCGCAAATGC-3′, probe 5′-AAGGCCTTCCTGGCCGATCCATC-3′.

Timeline of the experiments

Repeated systemic administration of PSY (0.1 mg/Kg; p.o.) every other day for two weeks

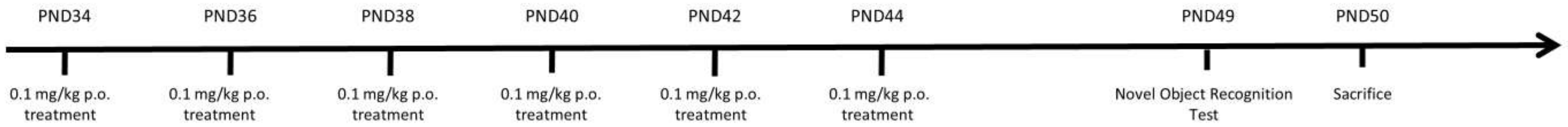

Pharmacological treatment schedule

Experiment N.	Treatment	Test / Analysis
1	*Systemic PSY + 5HT2A receptor antagonist M100 (0.25 mg/Kg; i.p.; 15 min before each oral PSY administration)*	*Novel Object Recognition Test*
2	*Systemic PSY + 5HT1A receptor antagonist WAY-100635 (0.3 mg/Kg; i.p.; 10 min before oral PSY administration)*	*Novel Object Recognition Test*
3	*Systemic PSY*	*Western blot analysis (Serotoninergic receptors)*
4	*Systemic PSY + TrkB receptor antagonist ANA-12 (0.5 mg/Kg; i.p.; 3 h and 30 min before oral PSY administration)*	*Novel Object Recognition Test*
5	*Systemic PSY + TrkB receptor antagonist ANA-12 (1.0 mg/Kg; i.p.; 3 h and 30 min before oral PSY administration)*	*Novel Object Recognition Test*
6	*Systemic PSY*	*qPCR analysis (BDNF)*
7	*Systemic PSY*	*Western blot analysis (Neurotrophic Factors + Downstream regulation)*

Supplementary Figure S1. Timeline of the experiments.

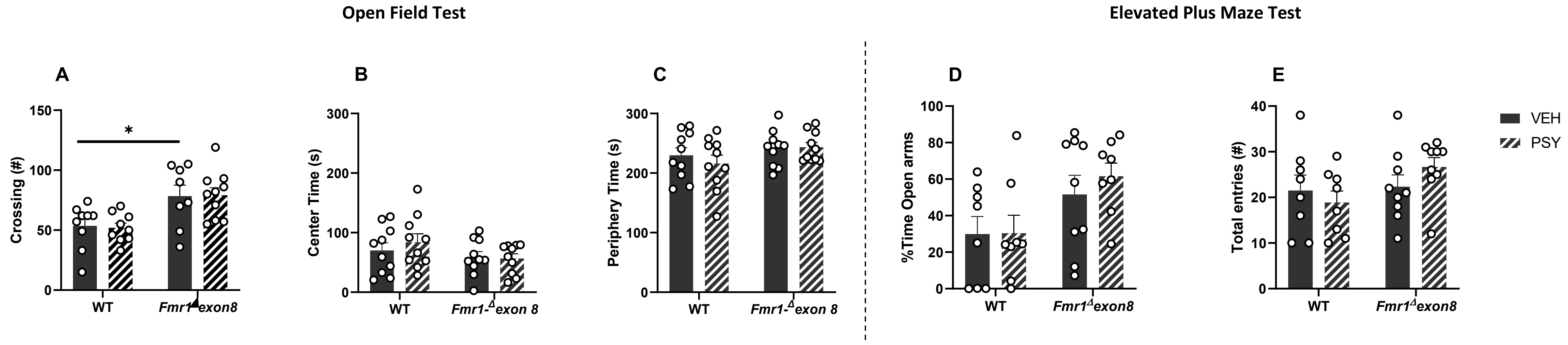

F

	Open Field Test								
	Crossing			Center Time			Periphery Time		
**ANOVA table**	**F (DFn, DFd)**	**P value**	**η²_p (partial eta squared)**	**F (DFn, DFd)**	**P value**	**η²_p (partial eta squared)**	**F (DFn, DFd)**	**P value**	**η²_p (partial eta squared)**
Genotype	F (1, 32) = 16,03	P=0,0003	0,33374974	F (1, 36) = 2,982	P=0,0928	0,076496845	F (1, 36) = 2,982	P=0,0928	0,076496845
PSY	F (1, 32) = 0,004143	P=0,9491	0,000129452	F (1, 36) = 0,2726	P=0,6048	0,007515315	F (1, 36) = 0,2726	P=0,6048	0,007515315
Genotype x PSY	F (1, 32) = 0,03631	P=0,8501	0,001133401	F (1, 36) = 0,4716	P=0,4967	0,012930609	F (1, 36) = 0,4716	P=0,4967	0,012930609

	Elevated Plus Maze Test					
	%Time Open arms			Total entries (#)		
**ANOVA table**	**F (DFn, DFd)**	**P value**	**η²_p (partial eta squared)**	**F (DFn, DFd)**	**P value**	**η²_p (partial eta squared)**
Genotype	F (1, 30) = 7,851	P=0,0088	0,207418562	F (1, 30) = 2,630	P=0,1153	0,080600674
PSY	F (1, 30) = 0,2343	P=0,6319	0,007749477	F (1, 30) = 0,1032	P=0,7503	0,003428207
Genotype x PSY	F (1, 30) = 0,1923	P=0,6642	0,006369174	F (1, 30) = 1,712	P=0,2007	0,053985873

Supplementary Figure S2. Behavioral assessment of locomotor and anxiety-like behaviors following psilocybin treatment in *Fmr1-$^{\Delta}$exon 8* rats. (A) Locomotor activity in the open field test, quantified as the number of crossings within a 5 min session (WT-VEH = 9, WT-PSY = 9, *Fmr1-$^{\Delta}$exon 8*-VEH = 8, *Fmr1-$^{\Delta}$exon 8*-PSY = 10). Time spent in the central (B) and peripheral (C) areas of the open field arena (WT-VEH = 10, WT-PSY = 10, *Fmr1-$^{\Delta}$exon 8*-VEH = 10, *Fmr1-$^{\Delta}$exon 8* - VEH= 10). Time spent in the open arms of the elevated plus-maze (D) and number of total entries (E) (WT-VEH = 8, WT-PSY = 8, *Fmr1-$^{\Delta}$exon 8*-VEH = 9, *Fmr1-$^{\Delta}$exon 8*-PSY = 8). (F) Summary of statistical analyses. Data represent mean ± SEM, *p < 0.05 vs WT-VEH group (two-way ANOVA followed by Tukey's post hoc test).

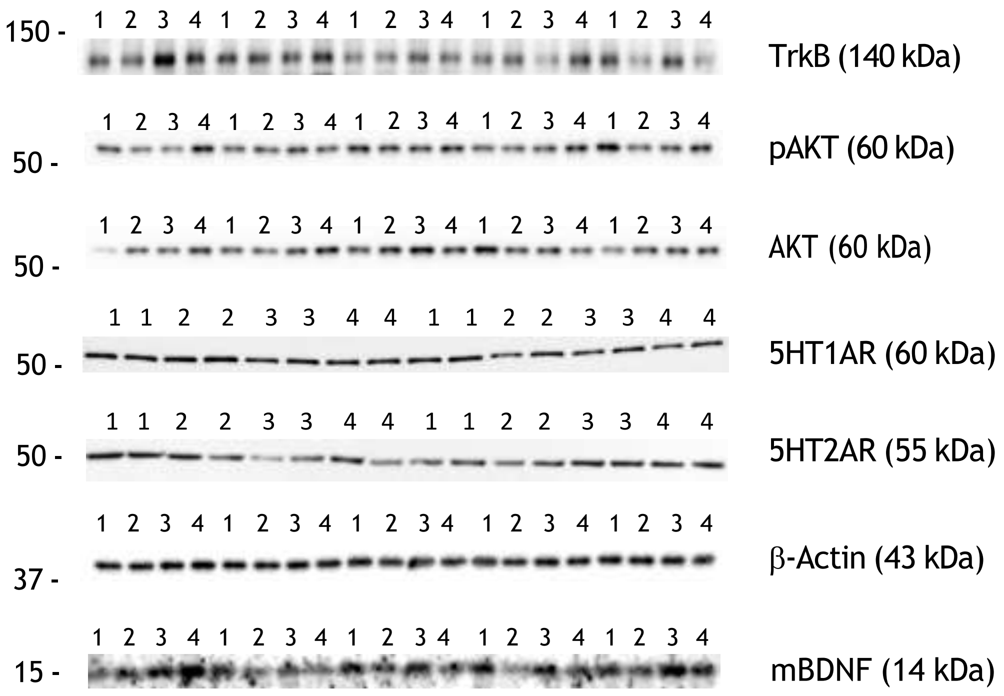

Supplementary Figure S3. Example of full-size cropped immunoblot related to the expression levels of TrkB (140 kDa), pAKT (60 kDa), AKT (60 kDa), 5HT1AR (60 kDa), 5HT2AR (55 kDa), b-Actin (43 kDa), mBDNF (14 kDa) measured in the whole homogenate fraction of the prefrontal cortex of wild-type vehicle-treated rats (1), *Fmr1-$^{\Delta}$exon 8* vehicle-treated rats (2), wild-type psilocybin-treated rats (3) and *Fmr1-$^{\Delta}$exon 8* psilocybin-treated rats (4).

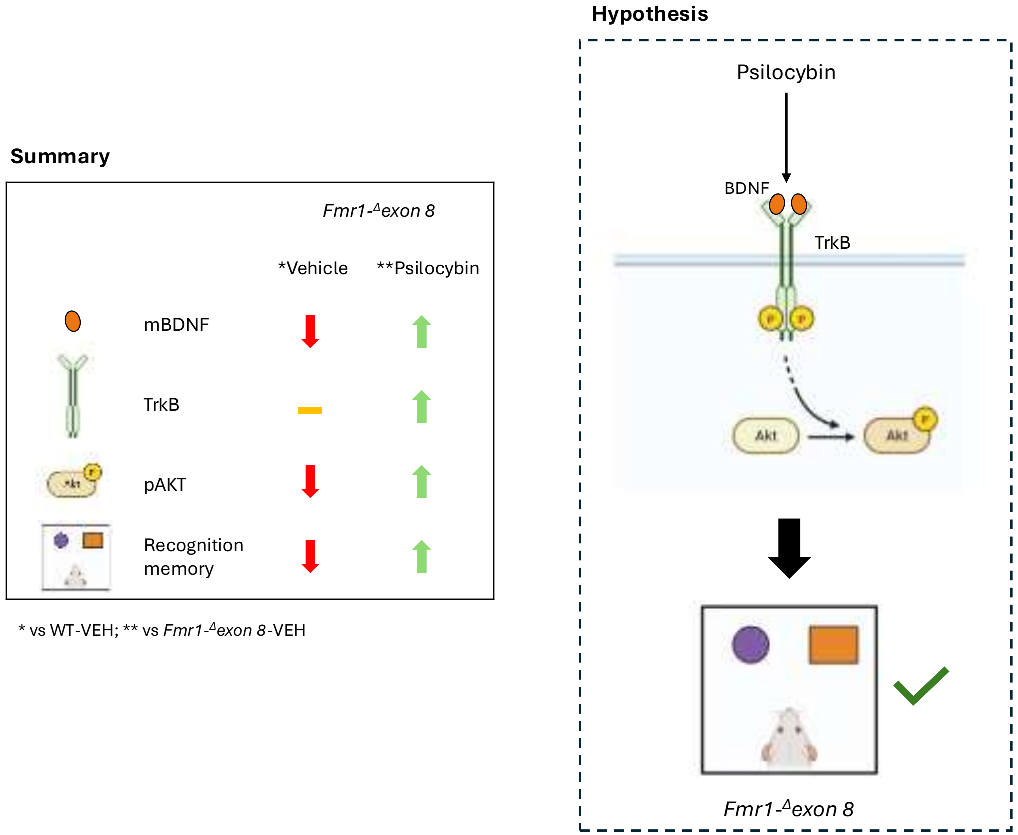

Supplementary Figure S4. Summary of main findings and proposed mechanistic framework.

	Experiment 1					
	Figure 1A			Figure 1B		
	% Discrimination index			Total time sniffing		
**ANOVA table**	**F (DFn, DFd)**	**P value**	**η²_p (partial eta squared)**	**F (DFn, DFd)**	**P value**	**η²_p (partial eta squared)**
Genotype	F (1, 66) = 12,57	P=0,0007	0,159984727	F (1, 66) = 0,8727	P=0,3536	0,013050168
M100	F (1, 66) = 0,06142	P=0,8050	0,000929741	F (1, 66) = 0,4936	P=0,4848	0,007423271
PSY	F (1, 66) = 11,95	P=0,0010	0,1533034	F (1, 66) = 0,1506	P=0,6992	0,002276623
Gen x M100	F (1, 66) = 1,282	P=0,2617	0,01905413	F (1, 66) = 0,04471	P=0,8332	0,000676966
Gen x PSY	F (1, 66) = 20,40	P<0,0001	0,236111111	F (1, 66) = 0,002448	P=0,9607	3,70895E-05
M100 x PSY	F (1, 66) = 0,1499	P=0,6999	0,002266065	F (1, 66) = 0,005503	P=0,9411	8,33718E-05
M100 x PSY x Gen	F (1, 66) = 0,6294	P=0,4304	0,00944628	F (1, 66) = 1,624	P=0,2070	0,024015143
	**Experiment 2**					
	**Figure 1C**			**Figure 1D**		
	**% Discrimination index**			**Total time sniffing**		
**ANOVA table**	**F (DFn, DFd)**	**P value**	**η²_p (partial eta squared)**	**F (DFn, DFd)**	**P value**	**η²_p (partial eta squared)**
Genotype	F (1, 59) = 11,43	P=0,0013	0,162288797	F (1, 59) = 6,773	P=0,0117	0,102975385
WAY	F (1, 59) = 1,793	P=0,1858	0,029493527	F (1, 59) = 2,433	P=0,1241	0,039604122
PSY	F (1, 59) = 4,950	P=0,0299	0,077404222	F (1, 59) = 0,2845	P=0,5958	0,004798893
Gen x WAY	F (1, 59) = 0,4514	P=0,5043	0,007592756	F (1, 59) = 0,1137	P=0,7372	0,001923412
Gen x PSY	F (1, 59) = 23,31	P<0,0001	0,283197667	F (1, 59) = 2,436	P=0,1239	0,039651019
WAY x PSY	F (1, 59) = 0,03329	P=0,8559	0,000563919	F (1, 59) = 0,3105	P=0,5795	0,005235161
WAY x PSY x Gen	F (1, 59) = 0,04631	P=0,8304	0,0007843	F (1, 59) = 0,0001746	P=0,9895	2,95931E-06

Supplementary Table 1

	Experiment 3					
	Figure 2A			Figure 2B		
	5HT2AR			5HT1AR		
	Protein levels (% vs WT-VEH)			Protein levels (% vs WT-VEH)		
**ANOVA table**	**F (DFn, DFd)**	**P value**	**η²_p (partial eta squared)**	**F (DFn, DFd)**	**P value**	**η²_p (partial eta squared)**
Gen x PSY	F (1, 12) = 3,350	P=0,0921	0,218241042	F (1, 12) = 1,124	P=0,3099	0,085644621
Genotype	F (1, 12) = 1,005	P=0,3359	0,07727797	F (1, 12) = 0,08767	P=0,7722	0,007252845
PSY	F (1, 12) = 2,804	P=0,1199	0,189408268	F (1, 12) = 6,819	P=0,0227	0,362346565

Supplementary Table 2

	Experiment 4					
	Figure 3A			Figure 3B		
	% Discrimination index			Total time sniffing		
**ANOVA table**	**F (DFn, DFd)**	**P value**	**η²_p (partial eta squared)**	**F (DFn, DFd)**	**P value**	**η²_p (partial eta squared)**
Genotype	F (1, 64) = 24,23	P<0,0001	0,274623144	F (1, 64) = 12,90	P=0,0006	0,167750325
ANA12 0.5	F (1, 64) = 3,325	P=0,0729	0,0493873	F (1, 64) = 0,03509	P=0,8520	0,000547981
PSY	F (1, 64) = 5,004	P=0,0288	0,072517535	F (1, 64) = 1,314	P=0,2559	0,020118198
Gen x ANA12 0.5	F (1, 64) = 2,806	P=0,0988	0,042002215	F (1, 64) = 0,0007256	P=0,9786	1,13374E-05
Gen x PSY	F (1, 64) = 16,77	P=0,0001	0,207626594	F (1, 64) = 0,6356	P=0,4282	0,00983359
ANA12 0.5 x PSY	F (1, 64) = 1,625	P=0,2070	0,024761905	F (1, 64) = 0,02354	P=0,8785	0,000367677
ANA12 0.5 x PSY x Gen	F (1, 64) = 0,1050	P=0,7469	0,001637938	F (1, 64) = 1,519	P=0,2223	0,023184115
	Experiment 5					
	Figure 3C			Figure 3D		
	% Discrimination index			Total time sniffing		
**ANOVA table**	**F (DFn, DFd)**	**P value**	**η²_p (partial eta squared)**	**F (DFn, DFd)**	**P value**	**η²_p (partial eta squared)**
Genotype	F (1, 60) = 71,62	P<0,0001	0,544142228	F (1, 60) = 0,8165	P=0,3698	0,013425633
ANA12 1	F (1, 60) = 1,225	P=0,2727	0,020008167	F (1, 60) = 0,6840	P=0,4115	0,011271505
PSY	F (1, 60) = 9,696	P=0,0028	0,139118457	F (1, 60) = 0,6311	P=0,4301	0,01040885
Gen x ANA12 1	F (1, 60) = 3,832	P=0,0549	0,060032586	F (1, 60) = 0,06785	P=0,7954	0,001129556
Gen x PSY	F (1, 60) = 14,79	P=0,0003	0,19775371	F (1, 60) = 1,790	P=0,1860	0,028969089
ANA12 1 x PSY	F (1, 60) = 5,156	P=0,0268	0,079133157	F (1, 60) = 0,4655	P=0,4977	0,007698605
ANA12 1 x PSY x Gen	F (1, 60) = 1,130	P=0,2921	0,018485195	F (1, 60) = 0,001686	P=0,9674	2,80992E-05

Supplementary Table 3

	Experiment 6								
	Figure 4A			Figure 4B			Figure 4C		
	Bdnf tot			*Bdnf long*			*Bdnf exon I*		
	mRNA levels (% vs WT-VEH)			mRNA levels (% vs WT-VEH)			mRNA levels (% vs WT-VEH)		
**ANOVA table**	**F (DFn, DFd)**	**P value**	**η²_p (partial eta squared)**	**F (DFn, DFd)**	**P value**	**η²_p (partial eta squared)**	**F (DFn, DFd)**	**P value**	**η²_p (partial eta squared)**
Gen x PSY	F (1, 16) = 24,91	P=0,0001	0,60889758	F (1, 16) = 0,01488	P=0,9044	0,000929136	F (1, 16) = 6,471	P=0,0217	0,287971163
Genotype	F (1, 16) = 35,65	P<0,0001	0,690222652	F (1, 16) = 38,69	P<0,0001	0,707441946	F (1, 16) = 174,6	P<0,0001	0,916054565
PSY	F (1, 16) = 2,295	P=0,1493	0,12544411	F (1, 16) = 0,3013	P=0,5907	0,018483188	F (1, 16) = 26,14	P=0,0001	0,620313242

	Figure 4D			Figure 4E		
	Bdnf exon IV			*Bdnf exon VI*		
	mRNA levels (% vs WT-VEH)			mRNA levels (% vs WT-VEH)		
**ANOVA table**	**F (DFn, DFd)**	**P value**	**η²_p (partial eta squared)**	**F (DFn, DFd)**	**P value**	**η²_p (partial eta squared)**
Gen x PSY	F (1, 16) = 0,5101	P=0,4854	0,030896239	F (1, 16) = 4,476	P=0,0504	0,218597382
Genotype	F (1, 16) = 22,31	P=0,0002	0,582354477	F (1, 16) = 8,062	P=0,0118	0,335051118
PSY	F (1, 16) = 0,1534	P=0,7005	0,009496453	F (1, 16) = 8,707	P=0,0094	0,352410248

Supplementary Table 4

	Experiment 7								
	Figure 5A			Figure 5B			Figure 5C		
	Protein levels (% vs WT-VEH)			Protein levels (% vs WT-VEH)			Protein levels (% vs WT-VEH)		
	mBDNF			TrkB			pAKT		
**ANOVA table**	**F (DFn, DFd)**	**P value**	**η²_p (partial eta squared)**	**F (DFn, DFd)**	**P value**	**η²_p (partial eta squared)**	**F (DFn, DFd)**	**P value**	**η²_p (partial eta squared)**
Gen x PSY	F (1, 16) = 6,946	P=0,0180	0,302710712	F (1, 16) = 10,36	P=0,0054	0,393019727	F (1, 16) = 27,73	P<0,0001	0,634118454
Genotype	F (1, 16) = 5,103	P=0,0382	0,24181396	F (1, 16) = 3,096	P=0,0976	0,162128194	F (1, 16) = 0,8082	P=0,3820	0,048083673
PSY	F (1, 16) = 4,485	P=0,0502	0,218940688	F (1, 16) = 3,729	P=0,0714	0,1890111	F (1, 16) = 0,001833	P=0,9664	0,000114549

	Figure 5D			Figure 5E		
	Protein levels (% vs WT-VEH)			Protein levels (% vs WT-VEH)		
	AKT			$pAKT_{s473}$/AKT		
**ANOVA table**	**F (DFn, DFd)**	**P value**	**η²_p (partial eta squared)**	**F (DFn, DFd)**	**P value**	**η²_p (partial eta squared)**
Gen x PSY	F (1, 16) = 4,141	P=0,0588	0,205600516	F (1, 16) = 37,39	P<0,0001	0,700318412
Genotype	F (1, 16) = 103,5	P<0,0001	0,866108787	F (1, 16) = 5,286	P=0,0353	0,248332237
PSY	F (1, 16) = 25,88	P=0,0001	0,617956065	F (1, 16) = 4,221	P=0,0566	0,208743386

Supplementary Table 5
